# Vulvar cancer in hidradenitis suppurativa

**DOI:** 10.1016/j.gore.2022.100929

**Published:** 2022-01-17

**Authors:** T.F.M. Vergeldt, R.J.B. Driessen, J. Bulten, T.H.J. Nijhuis, J.A. de Hullu

**Affiliations:** a Department of Obstetrics and Gynecology, Radboud University Medical Center, Geert Grooteplein Zuid 10, 6525 GA Nijmegen, the Netherlands; b Department of Dermatology, Radboud University Medical Center, Geert Grooteplein Zuid 10, 6525 GA Nijmegen, the Netherlands; c Department of Pathology, Radboud University Medical Center, Geert Grooteplein Zuid 10, 6525 GA Nijmegen, the Netherlands; d Department of Plastic and Reconstructive Surgery, Radboud University Medical Center, Geert Grooteplein Zuid 10, 6525 GA Nijmegen, the Netherlands

**Keywords:** Hidradenitis suppurativa, Squamous cell carcinoma, Vulvar cancer

## Abstract

**Background:**

Hidradenitis suppurativa (HS) is a debilitating disorder characterized by chronic inflammation in intertriginous areas. Malignant transformation to squamous cell carcinoma (SCC) is rare and is mostly diagnosed in the perianal area in men. The clinical behavior of SCC in HS can be aggressive, with local invasion and distant metastases.

Case descriptions.

We describe two cases of vulvar SCC in HS. The first demonstrates a 75 year old woman with a severe undertreated HS for over 30 years, who presented with a widespread vulvar cancer with lymphangitis carcinomatosa and inguinal and pelvic lymphadenopathy within several weeks after first suspicion of a malignancy. She died shortly after diagnosis. The second case describes a 61 year old woman diagnosed with HS 7 years ago, who presented with a rapidly progressive vulvar cancer with suspicion for ingrowth in the anal sphincter, vagina and levator ani muscle with inguinal and pelvic lymphadenopathy. She received radical chemoradiation with a complete response on imaging, but had a local recurrence within 2 months after finishing treatment. A posterior exenteration was performed but 5 months after surgery she had a second recurrence in the vulvar scar and pelvic floor muscles with possible bone metastases. She received palliative chemotherapy.

**Conclusion:**

Vulvar SCC in an area of HS is a rare condition which is difficult to diagnose. It can have an aggressive course with rapid progression and a high frequency of metastases at presentation. Early surgical excision of HS to diagnose occult malignant transformation, appropriate imaging to establish the extent of the disease and an aggressive treatment plan without any delays are recommended.

## Highlights

• Squamous cell carcinoma in hidradenitis suppurativa is difficult to diagnose.
• Early excision to diagnose occult malignancy is recommended.
• Vulvar cancer in hidradenitis suppurativa has a high frequency of metastases.
• Appropriate imaging to establish the extent of the disease is required.
• It has an aggressive course with rapid progression.

## 1 Introduction

Hidradenitis suppurativa (HS) is a chronic inflammatory disorder characterized by deep-seated nodules, abscesses, fistulae, suppurating sinus tracts, and scars in intertriginous areas, with a significant impact on quality of life (Goldburg et al., 2020). The prevalence in the general US and European population is estimated around 1% (Nguyen et al., 2021). HS has a strong association with obesity and smoking and it is likely that there is a genetic predisposition as 30–40% of patients with HS have at least one family member with the disease (Goldburg et al., 2020). HS is generally treated by a dermatologist using surgical interventions and/or anti-inflammatory agents, depending on the Hurley stage and the clinical phenotype (Horváth et al., 2017).

Pathogenesis involves immune dysregulation leading to increased secretion of antimicrobial peptides and inflammatory cytokines. Follicular hyperkeratosis and plugging leads to occlusion and dilatation of the pilosebaceous unit, with subsequent rupture and discharge of follicular content. This results in inflammatory reactions with an influx of neutrophils, lymphocytes and histiocytes leading to abscess formation and architectural tissue changes (Goldburg et al., 2020, Nguyen et al., 2021, Lavogiez et al., 2010).

Although HS is more common in women than in men, malignant transformation to squamous cell carcinoma (SCC) affects mainly men, mostly in the perianal or perineal area (Lavogiez et al., 2010, Jourabchi et al., 2017). The clinical behavior of SCC in HS can be aggressive, with local invasion and distant metastases.

We describe two cases of vulvar SCC in HS to increase awareness of this potential serious complication in patients with HS.

## 2 Case description 1

A 75 year old woman was referred to the dermatology department because of a severe undertreated HS Hurley stage III. Since her forties she had multiple surgical deroofings. She smoked 5 cigarettes a day.

She reported a continuous pain in her groins and labia with recurrent infection and bleeding. Physical examination showed multiple sinus tracts with suppurating nodules and abscess formation in her groins, vulvar and pubic region and perianally. On her left upper leg a lenticular erythematous livid exophytic papule located on a sinus tract was seen. A skin biopsy showed epidermal hyperplasia and an extensive chronic active inflammatory infiltrate without any signs of dysplasia or malignancy. She was prescribed clindamycin 300 mg twice daily with rifampicin 300 mg twice daily after which the inflammation regressed and the pain disappeared. As the inflammation and pain recurred after attempts to stop the antibiotics this treatment protocol was continued with satisfaction.

A few months later the patient mentioned that she had noticed a new lesion in her left groin. During physical examination extensive scarring, sinus tract formation, papules and comedones were observed as well as a 2 cm long fissure in her left groin without any signs of active infection. Differential diagnosis was a ruptured sinus tract in severe HS. Because of this observed progression despite treatment she was referred to a plastic surgeon for radical excision.

In the waiting time for the radical excision, the patient was reassessed by the dermatologist 2 months after the previous visit. The clinical presentation of the lesion in the left groin had changed with deroofing of the lesion and a yellowish debris. Differential diagnosis was granulation tissue or SCC. A biopsy revealed at least high grade dysplasia, but invasion could not be excluded. The surgical excision by the plastic surgeon was therefore expedited.

Three weeks after last inspection a radical excision of the HS in both groins was performed by the plastic surgeon. The patient was discharged a day later but required frequent returns to clinic because of compromised wound healing. Histology showed a SCC grade 3 in the left groin with depth of invasion of 11 mm without perineural or lymph vascular invasion. Excisional margin was positive for carcinoma. The histology of the right groin showed fistula and reactive changes but no dysplasia or carcinoma. A computed tomography (CT) scan of the chest and abdomen was performed with multiple enlarged inguinal lymph nodes and a 12 mm pelvic lymph node. Differential diagnosis was lymph node metastases or reactive lymphadenopathy postoperatively in a patient with active HS.

**Fig. 1:**
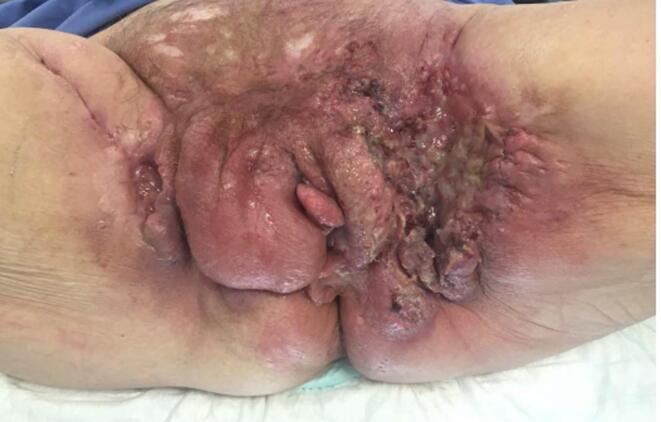

A re-excision was scheduled 6 weeks after the primary surgery. However, progression of the lesion in the left groin was visible with extensive vulvar changes suspicious for a widespread malignancy with lymphangitis carcinomatosa. The primary lesion was re-excised and vulvar mapping was performed (Fig. 1). Pathologic findings showed that all vulvar biopsies contained SCC grade 3 with lymph vascular invasion. Immunohistochemistry showed a p53 mutation without p16 expression and absence of high risk human papillomavirus (HPV) by Polymerase Chain Reaction (PCR).

Further treatment focused on best supportive care. Two weeks after discharge euthanasia was performed as per patient request because of refractory pain.

## 3 Case description 2

A 61 year old woman was diagnosed with HS around the age of 54. She had multiple surgical incisions and drainages because of her HS and was treated for a perianal abscess in the past. She smoked 5 cigarettes a day.

She complained of daily serosangulent and purulent fluid loss for which she used multiple gauzes and sanitary towels a day. This caused a lot of pain and fatigue and influenced her daily life. She used clindamycin 300 mg twice daily, without rifampicin because of side effects. Because this treatment was unsatisfactory in controlling her HS, screening for the start of adalimumab was initiated.

In the meantime she visited the dermatologist because of a painful vulvar lesion with purulent discharge and bleeding from this lesion. She had trouble sitting because of severe pain and had a weight loss of 6 kg in a few weeks. Physical examination showed an ulcer of 4–5 cm, and a palpable lymph node in the left groin of 2 cm. Vulvar biopsy showed a SCC grade 2 with lymph vascular invasion. Immunohistochemistry showed wildtype p53 and diffuse strong positive p16 expression. The PCR was positive for high risk HPV 16. Ultrasound guided biopsy of the inguinal lymph node did not confirm metastasis.

**Fig. 2:**
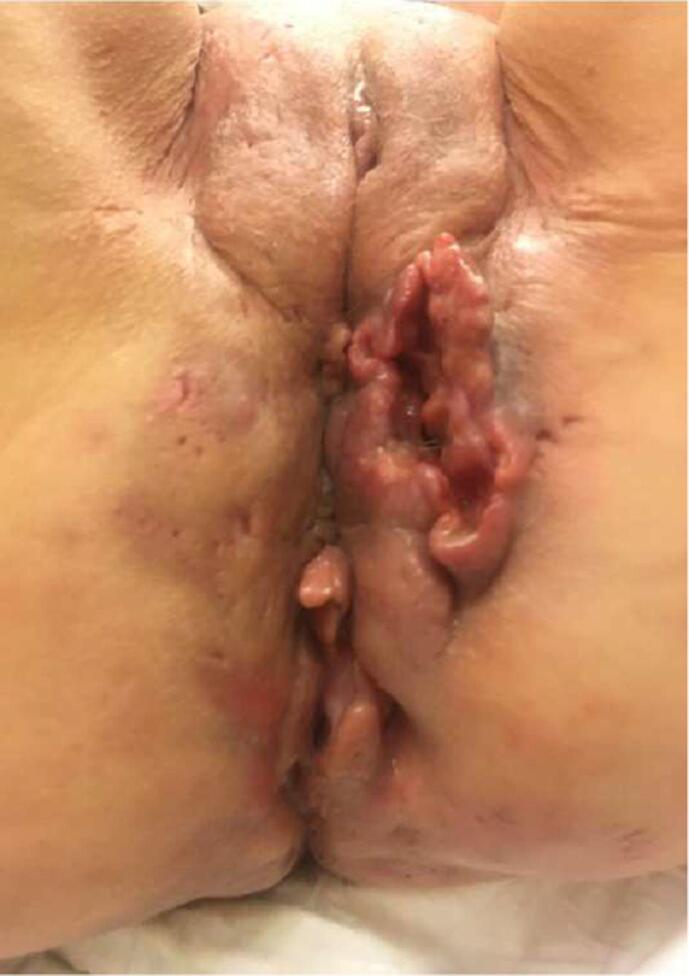

The patient was referred to a gynecologist. A joint consultation together with the radiotherapist was scheduled no later than 3 weeks following the last inspection. Physical examination showed an extensive ulcerating tumor of 7 by 4 cm and about 5–6 cm in depth, on the perineum, left and ventrally of the anus, with a second more solid lesion of 3 by 4 cm dorsally of the anus (Fig. 2). Pelvic magnetic resonance imaging (MRI) and a positron emission tomography-CT (PET-CT) scan showed a vulvar tumor with suspicion for ingrowth in the external anal sphincter, vaginal introitus and levator ani muscle with enlarged inguinal and pelvic lymph nodes.

She was treated with chemoradiotherapy 30 fractions of 1.65 Gy with a boost of 2.15 Gy on the vulvar tumor and suspicious lymph nodes, with weekly cisplatin 40 mg/m2. Because of side effects only 2 of the 5 scheduled cisplatin courses were administered. Post-treatment physical examination showed a complete response.

**Fig. 3:**
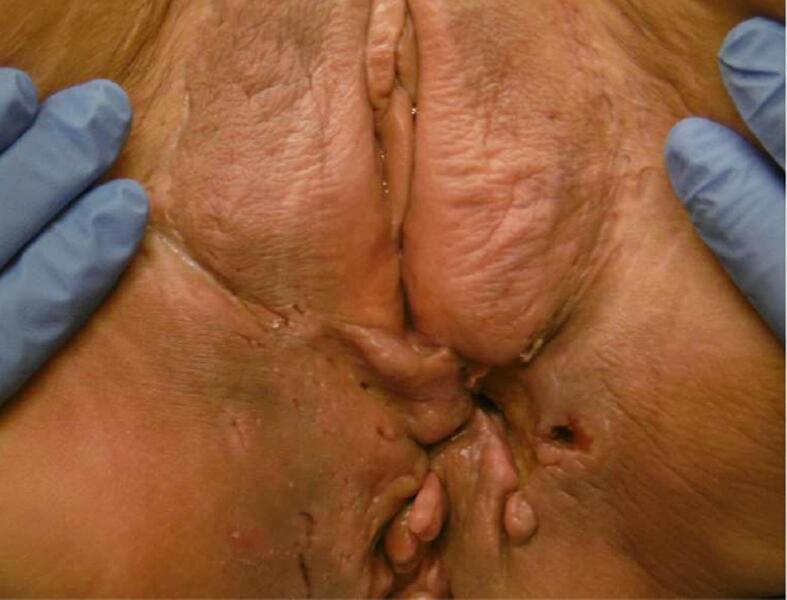

However, less than 2 months after treatment the pain recurred and a lesion of 2 by 3 cm was visualized in the same location as the primary tumor (Fig. 3). Biopsy confirmed the SCC. MRI and PET-CT showed a local recurrence in an area with extensive perianal fistula and inflammatory changes.

A posterior exenteration was performed including complete resection of the HS, the distal vagina, the anus and the rectosigmoid with a permanent colostomy. The wound was closed by a plastic surgeon using reconstruction with a Vertical Rectus Abdominis Muscle (VRAM) flap, a gluteal thigh flap, and a VAC pump. Pathology results revealed a SCC of 3.1 cm with a depth of invasion of 6.8 mm with free margins.

Physical and mental recovery was challenging. Five month after surgery, there was progressive vulvar pain with serosanguinolent fluid discharge. Inspection revealed residual HS with two small sinus tracts with induration and edema, but no visible or palpable signs of SCC recurrence. Nonetheless, MRI showed tumor recurrence in the vulvar scar and pelvic floor muscles with possible bone metastases. She received 6 cycles of palliative chemotherapy (carboplatin and paclitaxel) after which she had stable disease on imaging, but 3 months after completing the last cycle imaging showed progressive disease with a 6 cm tumor in the pelvic floor muscles with invasion of the pubic bone.

## 4 Discussion

These two cases show that vulvar SCC in an area of HS is difficult to diagnose and can have an aggressive course with rapid progression.

The various external features of HS make it difficult to identify SCC in HS. In a case series with 13 patients with SCC in HS, the clinical aspects of the suspect areas varied from a 3 cm ulcerating and granulating lesion to a 15–20 cm wide tumoral mass (Lavogiez et al., 2010). Superficial biopsies often only reveal an atypical pseudoepitheliomatous hyperplasia (Jourabchi et al., 2017). The occult spread of SCC along the subcutaneous sinus tracts makes it impossible to estimate the extent of the disease only by visual inspection. Early surgical excision of HS for histological diagnosis and appropriate imaging to establish the extent of the disease are highly recommended (Jourabchi et al., 2017).

Even though it is rare, there are several previously published case reports on women with SCC in an area of HS. Some of them describe patients with only stage 1 vulvar cancer with good outcome after surgery alone without adjuvant treatment (Rekawek et al., 2016, Sevray et al., 2019, Janse et al., 2017), however in line with the two cases in this current article there are several publications describing a more aggressive course and a high frequency of metastases at presentation. A review of literature on SCC in HS in 2010 found that almost half of the 38 patients developed lymph node metastases during follow-up compared to only 5–10% of patients with a SCC of the skin of any etiology (Lavogiez et al., 2010). In a case series of 13 patients, 2 patients had lymph node metastases and 3 patients had visceral metastases at presentation (Lavogiez et al., 2010). In a systematic review published in 2017, 7 cases of vulvar cancer and 6 cases of perineal/perianal carcinomas in patients with HS were included (Makris et al., 2017). Of the 7 patients with vulvar SCC, 3 had lymph node metastases or metastatic disease at presentation. Prognosis was poor. In comparison, in women with vulvar cancer in general about 40% have local lymph node metastasis at presentation, but distant metastases are rare (Klapdor et al., 2019, Oonk et al., 2006).

The association between HS and SCC is a topic of debate (Nguyen et al., 2021). Chronic inflammation probably plays a key role in the malignant transformation to SCC. A high stress atmosphere with tissue hyperplasia and hypertrophy leads to a high frequency of mutations. In combination with the dysregulation of tumor suppressor genes that co-occur with the inflammation, this produces an environment that favors oncogenesis (Chapman, 2018). The immune response generates free radicals leading to oxidative damage, which once more increases the risk for DNA mutations. Smoking is implicated as a risk factor, as tobacco smoke contains many mutagenic compounds that lead to an impaired immune function and mediate alterations in the normal balance between cell proliferation, differentiation and apoptosis, enhancing carcinogenesis (McBride et al., 2011). In a systematic review with 80 patients with SCC in HS, 80% of patients used tobacco (Jourabchi et al., 2017). A cocarcinogenic role of HPV infection in the malignant transformation of HS to SCC is postulated (Lavogiez et al., 2010, Jourabchi et al., 2017).

Intrinsically different subtypes of vulvar cancer have been identified (Kortekaas et al., 2020). The most common type is initiated by *TP53* mutations often in a background of lichen sclerosus, another is initiated by a high-risk HPV infection. With p53 and p16 immunohistochemistry the *TP53* mutational status respectively the HPV status can be determined to classify vulvar cancer into three molecular subtypes. In a retrospective cohort study with 415 women with vulvar cancer 18% of tumors were HPV positive, 66% of tumors were HPV negative with a p53 mutation and 15% of tumors were HPV negative with a p53 wildtype (Kortekaas et al., 2020). The women with a p53 mutation were more likely to have an advanced stage disease and had worse outcomes than women with HPV positive tumors or women who were HPV negative without a p53 mutation. In the two cases we presented the first woman had a negative HPV status and p53 was mutated, while in the second case HPV was positive and p53 was wildtype. This suggests that the HS itself, rather than the molecular subtype, affects the aggressive course of SCC in HS.

In conclusion, SCC and especially vulvar SCC in HS is a rare condition which is difficult to diagnose, with a high frequency of metastases at presentation and an aggressive course with rapid progression. We recommend early surgical excision of HS to diagnose occult malignant transformation, appropriate imaging prior to treatment to locate lymph node or distant metastases, and an aggressive treatment plan without any delays.

### CRediT authorship contribution statement

**T.F.M. Vergeldt:** Conceptualization, Writing – original draft. **R.J.B. Driessen:** Writing – review & editing. **J. Bulten:** Writing – review & editing. **T.H.J. Nijhuis:** Writing – review & editing. **J.A. de Hullu:** Writing – review & editing.

## Declaration of Competing Interest

The authors declare the following financial interests/personal relationships which may be considered as potential competing interests: Rieke Driessen reports grants and personal fees from Galderma, Novartis, Abbvie, Janssen and Leo Pharma, outside the submitted work. Fees were paid directly to the institution. The other authors have nothing to disclose.

## References

[b0005] Chapman S. (2018). Cutaneous squamous cell carcinoma complicating hidradenitis suppurativa: a review of the prevalence, pathogenesis, and treatment of this dreaded complication. Acta Dermatovenerol. Alp Pannonica Adriat..

[b0010] Goldburg S.R., Strober B.E., Payette M.J. (2020). Hidradenitis suppurativa: Epidemiology, clinical presentation, and pathogenesis. J. Am. Acad. Dermatol..

[b0015] Horváth B., Janse I., Blok J., Driessen R., Boer J., Mekkes J., Prens E., Zee H. (2017). Hurley Staging Refined: A Proposal by the Dutch Hidradenitis Suppurativa Expert Group. Acta Derm. Venereol..

[b0020] Janse I.D.G., Doff J., Mourits M., Horvath B. (2017). Hidradenitis Suppurativa: The Third Cause of Vulva Carcinoma. J. Clin. Obstet., Gynecol. Fertility.

[b0025] Jourabchi N., Fischer A.H., Cimino-Mathews A., Waters K.M., Okoye G.A. (2017). Squamous cell carcinoma complicating a chronic lesion of hidradenitis suppurativa: a case report and review of the literature. Int. Wound J..

[b0030] Klapdor R., Wölber L., Hanker L., Schmalfeldt B., Canzler U., Fehm T., Luyten A., Hellriegel M., Kosse J., Heiss C., Hantschmann P., Mallmann P., Tanner B., Pfisterer J., Jückstock J., Hilpert F., de Gregorio N., Hillemanns P., Fürst S.T., Mahner S. (2019). Predictive factors for lymph node metastases in vulvar cancer. An analysis of the AGO-CaRE-1 multicenter study. Gynecol. Oncol..

[b0035] Kortekaas K.E., Bastiaannet E., van Doorn H.C., de Vos van Steenwijk P.J., Ewing-Graham P.C., Creutzberg C.L., Akdeniz K., Nooij L.S., van der Burg S.H., Bosse T., van Poelgeest M.I.E. (2020). Vulvar cancer subclassification by HPV and p53 status results in three clinically distinct subtypes. Gynecol. Oncol..

[b0040] Lavogiez C., Delaporte E., Darras-Vercambre S., Martin De Lassalle E., Castillo C., Mirabel X., Laurent F., Patenotre P., Gheit T., Talmant J.C., Beylot-Barry M., Martinot V., Piette F., Aubin F., Mortier L. (2010). Clinicopathological study of 13 cases of squamous cell carcinoma complicating hidradenitis suppurativa. Dermatology.

[b0045] Makris G.-M., Poulakaki N., Papanota A.-M., Kotsifa E., Sergentanis T.N., Psaltopoulou T. (2017). Vulvar, Perianal and Perineal Cancer After Hidradenitis Suppurativa: A Systematic Review and Pooled Analysis. Dermatol. Surg..

[b0050] McBride P., Olsen C.M., Green A.C. (2011). Tobacco smoking and cutaneous squamous cell carcinoma: a 16-year longitudinal population-based study. Cancer Epidemiol. Biomarkers Prev..

[b0055] Nguyen T.V., Damiani G., Orenstein L.A.V., Hamzavi I., Jemec G.B. (2021). Hidradenitis suppurativa: an update on epidemiology, phenotypes, diagnosis, pathogenesis, comorbidities and quality of life. J. Eur. Acad. Dermatol. Venereol..

[b0060] Oonk M.H.M., Hollema H., De Hullu J.A., Van Der Zee A.G.J. (2006). Prediction of lymph node metastases in vulvar cancer: a review. Int. J. Gynecol. Cancer.

[b0065] Rekawek P., Mehta S., Andikyan V., Harmaty M., Zakashansky K. (2016). Squamous cell carcinoma of the vulva arising in the setting of chronic hidradenitis suppurativa: A case report. Gynecol. Oncol. Rep..

[b0070] Sevray M., Dupré P.-F., Le Flahec G., Trimaille A., Misery L., Brenaut E. (2019). Vulvar squamous cell carcinoma complicating hidradenitis suppurativa in a young woman. JAAD Case Rep..

